# M^3^-TransUNet: Medical Image Segmentation Based on Spatial Prior Attention and Multi-Scale Gating

**DOI:** 10.3390/jimaging12010015

**Published:** 2025-12-29

**Authors:** Zhigao Zeng, Jiale Xiao, Shengqiu Yi, Qiang Liu, Yanhui Zhu

**Affiliations:** 1 School of Computer and Artificial Intelligence, Hunan University of Technology, Zhuzhou 412007, China; 13617@hut.edu.cn (Z.Z.);; 2 School of Rail Transit, Hunan University of Technology, Zhuzhou 412007, China

**Keywords:** medical image segmentation, transunet, multi-scale, attention mechanism

## Abstract

Medical image segmentation presents substantial challenges arising from the diverse scales and morphological complexities of target anatomical structures. Although existing Transformer-based models excel at capturing global dependencies, they encounter critical bottlenecks in multi-scale feature representation, spatial relationship modeling, and cross-layer feature fusion. To address these limitations, we propose the M^3^-TransUNet architecture, which incorporates three key innovations: (1) MSGA (Multi-Scale Gate Attention) and MSSA (Multi-Scale Selective Attention) modules to enhance multi-scale feature representation; (2) ME-MSA (Manhattan Enhanced Multi-Head Self-Attention) to integrate spatial priors into self-attention computations, thereby overcoming spatial modeling deficiencies; and (3) MKGAG (Multi-kernel Gated Attention Gate) to optimize skip connections by precisely filtering noise and preserving boundary details. Extensive experiments on public datasets—including Synapse, CVC-ClinicDB, and ISIC—demonstrate that M^3^-TransUNet achieves state-of-the-art performance. Specifically, on the Synapse dataset, our model outperforms recent TransUNet variants such as J-CAPA, improving the average DSC to 82.79% (compared to 82.29%) and significantly reducing the average HD95 from 19.74 mm to 10.21 mm.

## 1. Introduction

In the realm of precision medicine, medical image segmentation serves as a fundamental component of medical image analysis. It facilitates the accurate extraction of anatomical structures or pathological regions across various imaging modalities—including X-ray, CT, and MRI—thereby playing a vital role in tumor lesion assessment, surgical pathway planning, and preoperative simulation for orthopedic or neurological procedures. However, anatomical structures are often characterized by notable inter-subject variability, while lesion manifestations evolve depending on the disease type and progression stage. For instance, ground-glass nodules in lung cancer CT scans typically present with blurred boundaries, whereas brain tumors frequently infiltrate surrounding normal tissue, resulting in indistinct margins. These complex characteristics present substantial challenges for segmentation algorithms. Traditional approaches, such as thresholding, region growing, and edge detection, may struggle when confronting subtle gray-scale variations and fuzzy boundaries. Consequently, they tend to yield over-segmented or under-segmented results, limiting their utility for high-precision automated segmentation tasks.

The integration of deep learning has facilitated the widespread adoption of convolutional neural networks (CNNs) [1] in medical image segmentation. Represented by U-Net [2], U-shaped architectures leverage symmetric encoder-decoder structures with skip connections to achieve multi-scale feature fusion. By effectively balancing low-level image details with high-level global semantics, this design has become a widely adopted paradigm. Subsequent variants, such as U-Net++ [3], U-Net3+ [4], and nnU-Net [5], have further refined feature integration strategies and cross-modal adaptability, improving segmentation accuracy and robustness. Furthermore, data-centric strategies—such as the interpolation-based augmentation proposed in [6]—have demonstrated observable improvements by enriching the diversity of training data. To bridge the gap between CNNs and Transformers [7], researchers initially introduced hybrid architectures like TransUNet [8], Swin-UNet [9], and CoTr [10], marking considerable progress in performance. More recently, advanced methods such as UNETR++ [11], TransBTS++ [12], and SegFormer3D [13] have further explored volumetric efficiency and long-range dependency modeling. Additionally, specialized architectures like MedNeXt [14], SAMed [15], and PolyFormer [16] have introduced novel mechanisms for enhanced contour delineation and semantic consistency. Despite these advancements, current paradigms still face limitations in the pursuit of superior accuracy: their multi-scale expressive capabilities could be further improved to support dynamic feature integration; their spatial modeling abilities are often constrained, as standard Transformers lack inherent spatial inductive biases, which impedes the capture of local anatomical nuances; and feature fusion across layers may result in semantic conflicts, where unscreened skip connections are prone to propagating noise, thereby affecting boundary precision.

To address these challenges, this paper proposes a high-performance hybrid segmentation network, termed M^3^-TransUNet, which aims to achieve competitive segmentation accuracy through refined multi-scale feature representation and advanced spatial relationship modeling. The primary contributions of this work are summarized as follows:

- To enhance multi-scale feature representation, we introduce MSGA in the encoder to adaptively weight and highlight the most informative scale-specific features. Concurrently, MSSA is deployed in the decoder to selectively integrate global semantic context from deep encoder features with fine-grained local details propagated via skip connections, enabling the precise reconstruction of object boundaries.
- Conventional self-attention mechanisms treat all pixel pairs impartially, neglecting their inherent geometric proximity. To mitigate this, we propose ME-MSA, which injects Manhattan distance as an additive spatial bias into the attention logits. This mechanism encourages the model to assign higher attention weights to spatially adjacent regions, thereby reinforcing local structural continuity while preserving global context awareness.
- The direct transmission of encoder features through skip connections frequently introduces irrelevant low-level patterns (e.g., texture noise or artifacts) that may degrade boundary precision. To address this, we propose MKGAG. This module refines skip connections by leveraging multi-kernel convolutions with diverse receptive fields to capture complementary spatial cues, followed by a channel-wise gating mechanism that suppresses non-essential components. This ensures that primarily semantically relevant and boundary-aware features are delivered to the decoder.

## 2. Related Work

### 2.1. CNN Skip Connections and Semantic Discrepancies

The U-Net-based encoder-decoder architecture has gained widespread adoption in medical image segmentation by fusing deep semantic features with shallow spatial details through skip connections. Early approaches, such as SegNet [17], ResUNet [18], and DenseNet [19], attempted to mitigate semantic discrepancies through structural innovations. However, traditional skip connections frequently falter due to the semantic incompatibility between shallow and deep features, as well as susceptibility to noise propagation. Task-specific adaptations have also been explored; for instance, Cheung et al. [20] proposed a computationally efficient U-Net variant for aorta and coronary artery segmentation that effectively balances accuracy and inference speed. To further address these issues, U-Net++ and U-Net3+ enhanced fusion through dense or full-scale connections, while Attention U-Net [21] employed attention gates to suppress irrelevant regions. Despite these advancements, these methods often struggle to adapt to complex scenarios with high variability.

To mitigate these limitations, we introduce the MKGAG module. By generating robust spatial attention maps through parallel multi-kernel grouped convolutions, it suppresses irrelevant noise from multiple angles while accentuating critical boundaries and details, thereby significantly enhancing the quality of feature fusion.

### 2.2. CNN-Transformer Hybrids in Medical Segmentation

In recent years, architectures represented by the Vision Transformer (ViT) [22] have been increasingly applied to medical image segmentation due to their superior global context modeling capabilities. Typical approaches include MedT [23], which introduces gated attention mechanisms, and the Swin Transformer [24], which utilizes hierarchical window-based self-attention to enhance computational efficiency. These works laid the foundation for subsequent hybrid architectures such as TransUNet and Swin-UNet. By synergizing the strong local feature extraction of CNNs with the long-range dependency modeling of Transformers, these hybrid models have achieved significant improvements in segmentation accuracy. However, while standard Transformer encoders excel at global modeling, their fixed tokenization strategies and lack of spatial continuity often disrupt the perception of fine-grained anatomical structures, such as vascular networks or tumor boundaries. Furthermore, vanilla self-attention treats all spatial positions impartially, lacking the image-specific inductive biases that are crucial for medical image understanding.

To address these issues, we propose a dual attention enhancement mechanism. First, we dynamically amplify critical local feature responses via the MSGA module prior to self-attention computation, enabling the model to prioritize informative details before engaging in global reasoning. Second, we integrate Manhattan distance-based spatial decay weights [25] into the multi-head self-attention mechanism. This introduces an explicit locality prior: pixels that are spatially closer are assigned higher initial attention weights, thereby encouraging the model to preserve local contextual coherence and enhancing its ability to capture boundary continuity and fine structural details.

### 2.3. Feature Fusion in Hybrid Architectures

In hybrid CNN-Transformer architectures, the efficient integration of heterogeneous feature types remains a significant challenge. Existing approaches predominately adopt the upsampling and concatenation strategy from U-Net, as seen in TransUNet, Swin-UNet, and UCTransNet [26]. However, naïve concatenation often results in high-resolution CNN features being overshadowed by the subsampled semantic information from the Transformer, leading to blurred boundaries and a loss of fine details. Previous studies such as CoTr, LeViT-UNet [27], MISSFormer [28], and MT-UNet [29] have attempted to mitigate this issue through efficient fusion schemes or multi-scale modeling, yet they still fall short in preserving sharp edges and intricate anatomical details.

To overcome this limitation, we propose the MSSA module. It extracts multi-scale local features through parallel multi-branch convolutions and integrates linear attention (specifically, PerformerAttention) to facilitate deep global-local interaction directly on high-resolution feature maps. This design provides precise, content-aware guidance that supersedes crude feature concatenation, ensuring more accurate segmentation—particularly for object boundaries and fine structural details.

## 3. Materials and Methods

### 3.1. Experimental Settings and Protocols

In this section, we detail the datasets, data augmentation strategies, implementation specifics, loss functions, and evaluation metrics used to validate the proposed M^3^-TransUNet.

#### 3.1.1. Datasets and Preprocessing

To comprehensively evaluate the segmentation performance and generalization capabilities of our model, we conducted experiments on four publicly available datasets covering distinct medical imaging modalities.

Synapse Multi-Organ Dataset. The primary evaluation was performed on the Synapse dataset from the MICCAI 2015 Multi-Modality Abdominal Annotation Challenge. This dataset comprises 30 abdominal CT scans, encompassing 3779 enhanced axial slices with a resolution of 512×512 pixels. The voxel spacing ranges from $\left[0.54∼0.54\right]\times \left[0.98∼0.98\right]\times \left[2.5∼5.0\right]\;{\mathrm{mm}}^{3}$. Annotations provided by professional radiologists cover eight abdominal organs: aorta, gallbladder, liver, pancreas, stomach, spleen, and the left and right kidneys. Following standard protocols, we adopted a random split of 18 cases for training and 12 cases for validation.

Generalization Benchmarks. To further validate model robustness, we extended experiments to three additional datasets:

(1) CVC-ClinicDB for polyp segmentation in colonoscopy, containing 612 images (384×288) characterized by significant lighting variations;
(2) Chest X-ray Masks and Labels, providing 704 grayscale chest X-rays for lung segmentation, challenging models with noise and occlusions; and
(3) ISIC 2016 for dermatological lesion segmentation, consisting of 900 dermoscopy images where lesion boundaries are often blurred.

Preprocessing. For the Synapse dataset, image intensity values were truncated to the range of [−125,275] HU and normalized to [0,1]. For all datasets, images were resized to a uniform resolution of 224×224 pixels to meet the input requirements of the hybrid encoder.

#### 3.1.2. Data Augmentation Strategy

Given the limited scale of medical datasets, we employed an on-the-fly (online) data augmentation strategy during training to mitigate overfitting. Unlike offline expansion, this approach applies random transformations dynamically as data is loaded. Consequently, the effective number of samples per epoch corresponds to the original training set size, ensuring that the model encounters diverse variations over time without artificially inflating the dataset storage. The pipeline, implemented using Albumentations, includes:

- Geometric Transformations: Horizontal flipping (probability =0.5), random translation, scaling, and rotation ($\pm 20^{\circ }$) were applied to enhance invariance to spatial variations.
- Structural Transformations: Elastic deformation was used to mimic non-rigid soft tissue distortions, and random occlusion was applied to promote global contextual reasoning.
- Intensity Transformations: Random brightness/contrast adjustments, Gaussian noise, and CLAHE were employed to increase resilience to imaging artifacts and heterogeneous lighting.

Crucially, all data augmentation techniques were strictly restricted to the training phase. All quantitative evaluations reported in this study were conducted on the original, non-augmented test set to ensure the fairness and reliability of the results.

#### 3.1.3. Implementation Details

The framework was implemented in PyTorch v2.7.1 and trained on the Synapse dataset for 20,000 iterations. We employed the Stochastic Gradient Descent (SGD) optimizer with an initial learning rate of 0.01, a momentum of 0.9, and a weight decay of $1\times 10^{-4}$. The batch size was set to 24. Foundational experiments were conducted on a single NVIDIA RTX 4090 GPU, while large-scale validation utilized an NVIDIA A800 server.

#### 3.1.4. Loss Function Design

We employ a compound loss function combining Cross-Entropy and Dice Loss to leverage their complementary strengths.

(1) $$L_{\mathrm{total}}=\lambda_{1}L_{\mathrm{CE}}+\lambda_{2}L_{\mathrm{Dice}}$$

where $\lambda_{1}=\lambda_{2}=0.5$. This equal weighting strategy was determined empirically. Preliminary experiments indicated that this balance effectively stabilizes gradient descent by mitigating the gradient vanishing issue of Dice loss for small targets while preventing Cross-Entropy from dominating the optimization of large background regions.

Cross-Entropy Loss provides smooth gradients for pixel-level classification:

(2) $$L_{CE}=-\frac{1}{N}\sum_{i=1}^{N}\sum_{c=1}^{C}y_{i,c}\log\left(p_{i,c}\right)$$

Dice Loss [30] directly optimizes the overlap metric and mitigates class imbalance:

(3) $$L_{\mathrm{Dice}}=1-\frac{1}{C-1}\sum_{c=1}^{C-1}\frac{2\sum_{i=1}^{N}p_{i,c}y_{i,c}}{\sum_{i=1}^{N}p_{i,c}+\sum_{i=1}^{N}y_{i,c}}$$

#### 3.1.5. Evaluation Metrics

Segmentation performance is assessed using two complementary metrics: Dice Similarity Coefficient (DSC) measures the regional overlap:

(4) $$DSC=\frac{2\times |P\cap G|}{\left|P|+|G\right|}$$

Hausdorff Distance (HD95) evaluates boundary accuracy by measuring the 95th percentile of the maximum distances between predicted and ground truth boundaries:

(5) $$HD\left(P,G\right)=\max\left\{h(P,G),\;h(G,P)\right\}$$

Lower HD95 values indicate better boundary adherence.

### 3.2. Network Architecture

#### 3.2.1. Overall Architecture

**Figure 1:**
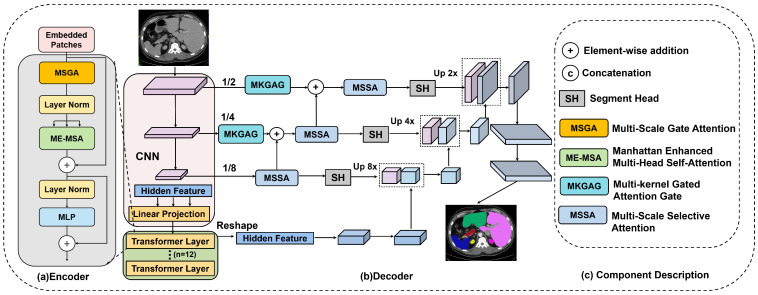
Overview of the M^3^-TransUNet architecture. (**a**) The hybrid encoder integrating MSGA and ME-MSA. (**b**) The progressive fusion decoder with MSSA. (**c**) Legend of specific components.

The proposed M^3^-TransUNet architecture leverages a hybrid U-shaped framework, as illustrated in Figure 1. This design integrates the local feature extraction capabilities of CNNs with the global dependency modeling of Transformers. By effectively balancing fine-grained boundary delineation with holistic semantic understanding, the model achieves enhanced accuracy in medical image segmentation tasks.

The workflow begins with a multi-scale CNN module that employs varying receptive fields to extract hierarchical features from the input image. To ensure compatibility between the grid-based CNN and the sequence-based Transformer, we implement specific dimension alignment operations. As shown in Figure 1a, the extracted feature maps are flattened and linearly projected into a sequence representation to match the embedding dimension, then fed into a 12-layer stacked encoder. Conversely, before entering the decoder, the hidden feature sequence is reshaped back into spatial dimensions.

Each encoder layer incorporates two key components: the MSGA and the ME-MSA modules. Within an encoder block, input features sequentially traverse the MSGA, LN, and ME-MSA. The output of ME-MSA is refined via a residual connection [31], followed by a second LN and an MLP. A final residual connection adds the MLP output to its input, yielding the updated feature representation.

Crucially, these encoded features serve a dual purpose: they are propagated to subsequent encoder layers to extract deeper semantics and transmitted as hidden features to the decoder via skip connections. To resolve potential feature discrepancies between the encoder and decoder, these connections are mediated by the MKGAG. Regarding optimization, the entire framework consists exclusively of differentiable operators, enabling end-to-end training. A unified loss function drives gradient propagation through both the CNN and Transformer components simultaneously, ensuring that all blocks are jointly optimized.

#### 3.2.2. Multi-Scale Feature Enhancement via MSGA

**Figure 2:**
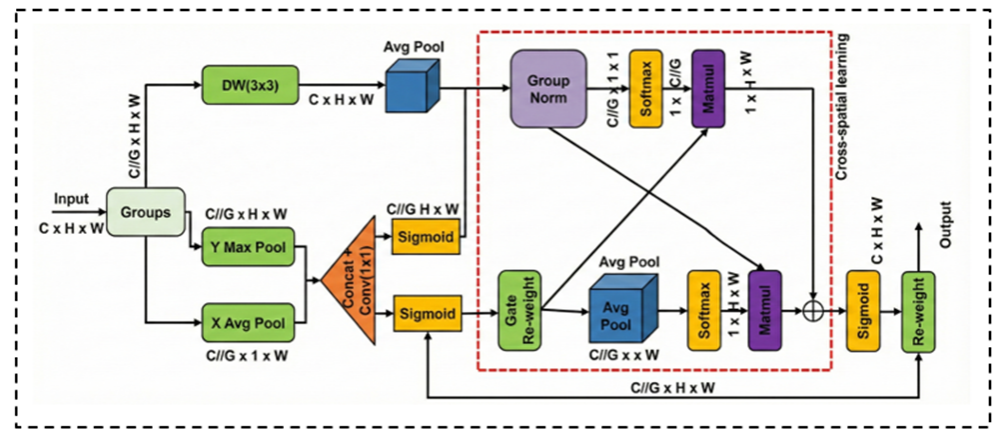
Architecture of the Multi-Scale Gated Attention (MSGA) module.

In standard Transformer encoders, token-based processing often neglects channel-wise dependencies and local scale variations. To address these limitations, we propose the MSGA module. As illustrated in Figure 2, unlike conventional parallel designs, MSGA adopts a cascaded feature modulation strategy to progressively refine local and global contexts.

The input feature $X\in R^{C\times H\times W}$ is first partitioned into *G* non-overlapping subgroups [31] along the channel dimension. For each subgroup $X^{g}$, the processing pipeline consists of two sequential stages:

- Local Feature Modulation: The first stage captures local semantics and suppresses background noise through a dual-path mechanism. The upper path utilizes a 3×3 Depthwise Convolution (DW) to extract fine-grained structural details. Simultaneously, the lower path employs a coordinate attention strategy to generate a spatial gating map. Specifically, we apply average pooling and max pooling along horizontal and vertical directions to capture long-range dependencies. The pooled features are concatenated and fused via a 1×1 convolution to produce a spatial attention map $M_{sp}^{g}$. This map modulates the depthwise features $F_{dw}^{g}$ via element-wise multiplication:
  (6) $$F_{dw}^{g}={\mathrm{DWConv}}_{3\times 3}\left(X^{g}\right),$$
  (7) $$M_{sp}^{g}=\sigma \left({\mathrm{Conv}}_{1\times 1}\left(\mathrm{Concat}\left(\mathrm{AvgPool}\left(X^{g}\right),\mathrm{MaxPool}\left(X^{g}\right)\right)\right)\right),$$
  (8) $$F_{gated}^{g}=F_{dw}^{g}⊙M_{sp}^{g},$$
  where σ denotes the Sigmoid function and ⊙ represents element-wise multiplication.
- Cross-Spatial Interaction and Fusion: The gated feature $F_{gated}^{g}$ is then fed into the Cross-Spatial Learning block (dashed box in Figure 2) to model global correlations. This block comprises two interacting streams: one utilizes Group Normalization to preserve feature distribution, while the other employs Average Pooling to abstract global context. These streams interact via matrix multiplication to exchange spatial and channel information. The outputs of these dual streams are fused via element-wise addition to integrate complementary contexts. Finally, the aggregated feature map passes through a Sigmoid activation to generate the final re-weighting mask. This mask is applied to the original subgroup input $X^{g}$ via a residual-style re-weighting operation to yield the enhanced output $Y^{g}$:
  (9) $$Y^{g}=X^{g}⊙\sigma \left(\mathrm{CrossSpatial}(F_{gated}^{g})\right).$$

This formulation explicitly combines the local texture details preserved by the input with the refined global semantic context, ensuring precise boundary delineation.

#### 3.2.3. Spatial-Aware Modeling with ME-MSA

Standard self-attention treats all tokens equally regardless of their spatial distance, often suffering from “attention spillover,” where irrelevant distant regions (e.g., background artifacts) distract from local anatomical details. To address this, we propose the ME-MSA, which injects a geometric prior into the attention mechanism.

Unlike Euclidean distance, Manhattan distance aligns better with the grid-like structure of digital images. We incorporate this prior as a learnable attention bias. Specifically, for any two tokens *i* and *j* with spatial coordinates $\left(x_{i},y_{i}\right)$ and $\left(x_{j},y_{j}\right)$, their normalized Manhattan distance $D_{ij}$ is computed as:

(10) $$D_{ij}=\frac{|x_{i}-x_{j}\left|+\right|y_{i}-y_{j}|}{H+W}$$

where *H* and *W* are the height and width of the feature map.

Instead of a hard mask, we transform this distance into a learnable continuous bias term $B_{ij}$. This bias is subtracted from the scaled dot-product attention logits to penalize long-range dependencies adaptively:

(11) $$\mathrm{Attention}\left(Q,K,V\right)=\mathrm{Softmax}\left(\frac{QK^{⊤}}{\sqrt{d_{k}}}-\lambda \cdot D\right)V$$

where λ is a learnable scalar initialized to 1.0, allowing the network to automatically adjust the strength of the spatial constraint during training. By explicitly prioritizing spatially adjacent features, ME-MSA preserves local structural continuity—a critical requirement for delineating lesion boundaries.

Validation of the Manhattan Prior: While the ME-MSA is integrated with MSGA within the encoder blocks, its specific contribution to performance can be inferred from the ablation results. Standard self-attention mechanisms often lack spatial inductive biases, leading to fragmented boundaries. In our study, the integration of the encoder module resulted in a marked reduction in boundary error (HD95 dropped from 19.33 mm to 11.48 mm), as detailed in the ablation studies within Section 4.2.5. Since MSGA primarily targets multi-scale feature representation, this substantial improvement in boundary precision is inherently driven by the Manhattan spatial prior of ME-MSA, which effectively constrains the attention mechanism to prioritize spatially contiguous anatomical structures.

#### 3.2.4. Gated Skip Connections with MKGAG

**Figure 3:**
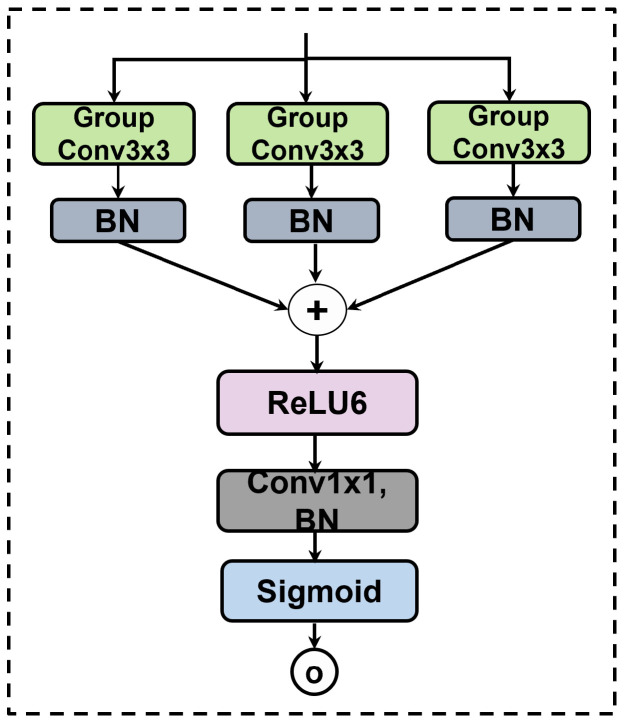
Architecture of the Multi-Kernel Gated Attention Gate (MKGAG) module.

Naive skip connections often transfer irrelevant low-level noise directly to the decoder. To achieve dynamic feature selection, we propose the MKGAG, illustrated in Figure 3. Its core function is to refine features by suppressing background noise through an ensemble-like feature aggregation mechanism.

The MKGAG module operates on the skip-connected features $X\in R^{C\times H\times W}$ through two key steps:

- Multi-Branch Feature Extraction: To enrich feature diversity and capture complementary semantic information, we employ a parallel multi-branch architecture. The input X is processed simultaneously by three independent branches. Each branch consists of a 3×3 Group Convolution followed by Batch Normalization (BN) [32,33,34]. By utilizing independent learnable parameters for each branch, this design mimics an ensemble of local feature extractors, enabling the model to learn diverse representations from the same spatial context. The outputs of the three branches, denoted as $H_{1},H_{2},H_{3}$, are formulated as:
  (12) $$H_{k}=\mathrm{BN}\left({\mathrm{GroupConv}}_{3\times 3}^{\left(k\right)}\left(X\right)\right),\;k\in \left\{1,2,3\right\}$$
  where ${\mathrm{GroupConv}}^{\left(k\right)}$ denotes the *k*-th group convolution operation with independent weights.
- Gating Signal Generation and Refinement: To generate the attention weights, the features from the three branches are aggregated via element-wise summation. The fused feature map passes through a ReLU6 activation [35] to introduce non-linearity, followed by a 1×1 convolution to compress the channel dimension to 1. Finally, a Sigmoid function generates the spatial attention map $A\in R^{1\times H\times W}$. The final output Y is obtained by modulating the original input X with this attention map:
  (13) $$A=\sigma \left({\mathrm{Conv}}_{1\times 1}\left(\mathrm{ReLU}6\left(\sum_{k=1}^{3}H_{k}\right)\right)\right)$$
  (14) Y=X⊙A
  where σ is the Sigmoid activation and ⊙ represents element-wise multiplication. This gating mechanism ensures that only semantically relevant regions are propagated to the decoder.

#### 3.2.5. Progressive Decoding with MSSA

**Figure 4:**
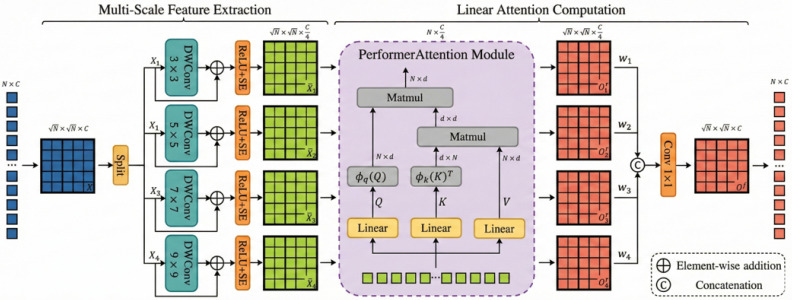
Architecture of the Multi-Scale Selective Attention (MSSA) module.

We propose a multi-scale progressive fusion decoder to accurately map deep semantic features to pixel-level predictions. The core of this decoder is the Multi-Scale Selective Attention (MSSA) module, shown in Figure 4.

The decoder follows a merge-augment-upsample paradigm. Low-resolution features are upscaled, combined with skip-connected features, and then refined by MSSA. This module operates in three synergistic stages:

- Multi-Scale Feature Extraction: Recognizing that anatomical structures vary drastically in size, we employ four parallel Depthwise Separable Convolution (DWConv) branches with kernel sizes k∈{3,5,7,9}. This multi-scale design captures diverse anatomical contexts—small kernels (3×3) focus on fine vessel boundaries, while larger kernels (9×9) capture broad organ contours. Furthermore, each branch employs a Squeeze-and-Excitation (SE) block to recalibrate channel-wise feature responses. The multi-scale representation $F_{ms}$ is obtained by concatenating these branches:
  (15) $$F_{ms}={\mathrm{Concat}}_{k\in \{3,5,7,9\}}\left(\mathrm{SE}(\mathrm{ReLU}\left({\mathrm{DWConv}}_{k\times k}\left(F_{in}\right)\right))\right)$$
- Efficient Linear Attention: To model long-range dependencies without the quadratic complexity of standard Transformers, features from all branches are fused and processed by a Performer Attention [36] layer. This mechanism approximates standard attention with linear complexity O(N) using random Fourier features. We project $F_{ms}$ into query (Q), key (K), and value (V) matrices. By utilizing a kernel approximation function ϕ(·), the global context is computed as:
  (16) $$\mathrm{Attention}\left(Q,K,V\right)=D^{-1}\left(\phi \left(Q\right)\left(\phi {\left(K\right)}^{⊤}V\right)\right),$$
  (17) $$D=\mathrm{diag}(\phi \left(Q\right)\phi {\left(K\right)}^{⊤}1)$$
  where D serves as the normalization term. This enables the model to maintain global context awareness even on high-resolution feature maps.
- Feature Fusion: The refined features from the attention layer are concatenated and integrated via a 1×1 convolution. This step restores the original channel dimension and ensures compatibility with downstream layers.

## 4. Results

### 4.1. Comparative Analysis with State-of-the-Art Methods

To validate the effectiveness of the proposed M^3^-TransUNet, we conducted a comprehensive evaluation on the Synapse multi-organ segmentation dataset. We benchmarked our model against a diverse set of baselines, including classical Convolutional Neural Networks (e.g., U-Net, U-Net++, Residual U-Net, Attention U-Net, and MultiResUNet [37]) and mainstream Transformer-based architectures (e.g., TransUNet, UCTransNet, TransNorm [38], Swin-UNet, DA-TransUNet [39], HiFormer-B [40], PVT-VASVADE [41], and J-CaPA [42]). Detailed quantitative comparisons are presented in Table 1.

#### 4.1.1. Segmentation Performance Analysis

As summarized in Table 1, M^3^-TransUNet demonstrates competitive performance on the Synapse dataset, achieving an average Dice Similarity Coefficient (DSC) of 82.79% and a 95% Hausdorff Distance (HD95) of 10.21 mm. These metrics exceed those of both traditional CNNs and advanced hybrid transformers. Furthermore, to validate the reliability of these improvements, we conducted a Wilcoxon signed-rank test. The results confirm that the performance gains of M^3^-TransUNet over the competitive baselines are statistically meaningful with p<0.05.

Specifically, compared to representative CNN-based methods such as U-Net (76.85%) and Att-U-Net (77.77%), our model improves the average DSC by approximately 5.9–6.0%, indicating enhanced capability in modeling complex anatomical variances. Moreover, it outperforms leading Transformer-based models—including TransUNet (77.48%), DA-TransUNet (79.80%), PVT-VASVADE (81.06%), and J-CaPA (82.29%)—confirming the efficacy of our multi-scale feature fusion and global-local semantic modeling strategy.

In terms of boundary delineation, M^3^-TransUNet achieves a notably low HD95 of 10.21 mm. This represents a reduction of approximately 25–30 mm compared to traditional CNN approaches (e.g., U-Net: 39.70 mm) and 7–17 mm compared to advanced Transformer counterparts (e.g., HiFormer-B: 14.70 mm), suggesting improved structural preservation and edge consistency.

Regarding organ-specific segmentation, M^3^-TransUNet delivers consistently robust results across all targets. Notably, it achieves 86.37% DSC for the left kidney (surpassing HiFormer-B’s 85.23%) and 83.08% for the right kidney (exceeding DA-TransUNet’s 80.45%). On the challenging pancreas segmentation task, our model attains 68.45% DSC, outperforming TransUNet (55.86%) by a margin of 12.59% and DA-TransUNet (61.62%) by 6.83%. Additionally, it achieves the highest DSC among all compared methods for both the spleen (90.88%) and the stomach (82.21%).

These results collectively indicate that M^3^-TransUNet is particularly effective at segmenting small organs and low-contrast anatomical structures with high precision.

#### 4.1.2. Computational Complexity and Efficiency Analysis

To evaluate the trade-off between segmentation accuracy and resource consumption, we compared the proposed M^3^-TransUNet with state-of-the-art methods regarding parameters (Params) and floating-point operations (FLOPs). The quantitative results are summarized in Table 2.

- Model Capacity and Computational Cost: As presented in Table 2, the proposed model utilizes 116.95 M parameters. We acknowledge that this parameter footprint exceeds that of lightweight transformers such as Swin-UNet (27.14 M). It is worth noting that a higher parameter count is often an inherent characteristic of hybrid ViT-based architectures (similar to the baseline TransUNet) that necessitate dense attention layers to capture global long-range dependencies. However, regarding computational speed (FLOPs), our model demonstrates competitive efficiency. Specifically, M^3^-TransUNet requires 32.08 G FLOPs, which is lower than the hybrid baseline TransUNet (38.52 G) and the standard U-Net (65.52 G). This indicates that while the architecture follows the capacity-rich design of ViT-based models, it optimizes the inference process by avoiding excessive redundant calculations.
- Performance Trade-off and Future Optimization: The increased model capacity contributes to improved segmentation accuracy, with M^3^-TransUNet achieving a DSC of 82.79%. This performance surpasses TransUNet by 5.31% and J-CaPA by 0.50%. The parameter overhead is primarily attributed to the multi-branch parallel convolutions in the MSSA and MKGAG modules, which are designed to capture fine-grained multi-scale anatomical details. Nevertheless, the high parameter count presents a limitation for deployment on resource-constrained edge devices. We recognize this as a key area for improvement. Consequently, our future work will prioritize lightweight optimization to reduce the model size while maintaining its high segmentation precision.

#### 4.1.3. Training Dynamics and Convergence Analysis

**Figure 5:**
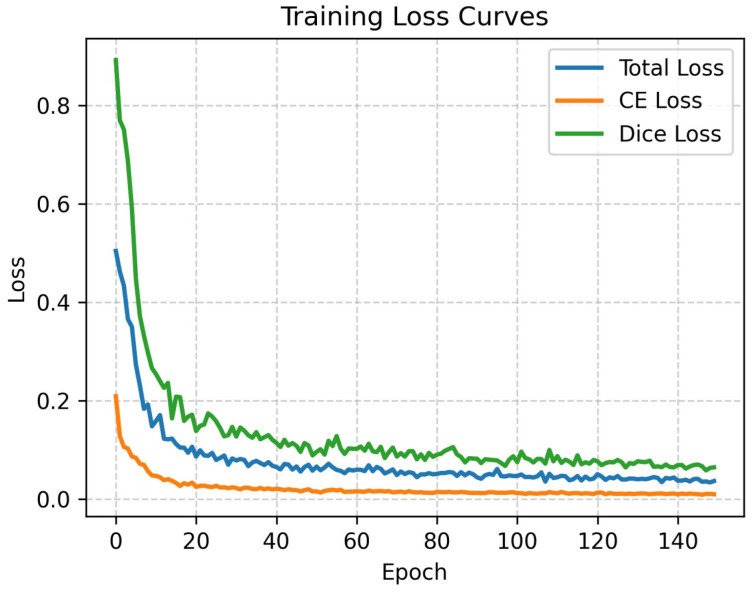
Training loss curves on the Synapse dataset. The plot displays the Total Loss, Cross-Entropy (CE) Loss, and Dice Loss over 150 epochs. The rapid initial decrease followed by a stable plateau demonstrates effective convergence.

To further substantiate the robustness of M^3^-TransUNet beyond the comparative metrics, we analyzed the training dynamics to verify its stability and convergence behavior. Figure 5 illustrates the progression of Total Loss, Cross-Entropy (CE) Loss, and Dice Loss over 150 epochs on the Synapse dataset.

It can be observed that the loss curves exhibit a sharp descent during the initial phase (0–20 epochs), indicating that the model efficiently learns feature representations, facilitated by the inductive biases inherent in the CNN branch. Subsequently, the curves transition into a smooth decline and stabilize around 100 epochs without significant oscillations or divergence. This behavior confirms that the optimization process is stable and that the proposed hybrid architecture converges effectively. Although this visualization primarily depicts training dynamics, the consistently competitive results on the unseen test set (as detailed in Table 1) provide strong empirical evidence that the model has learned robust features rather than simply memorizing the training data.

### 4.2. Ablation Studies

To rigorously evaluate the contribution of each component and the impact of hyperparameter settings, we conducted a series of ablation experiments focusing on: (1) skip connections, (2) input resolution, (3) patch size, (4) model scale, and (5) architectural modules.

#### 4.2.1. Impact of Skip Connections

**Figure 6:**
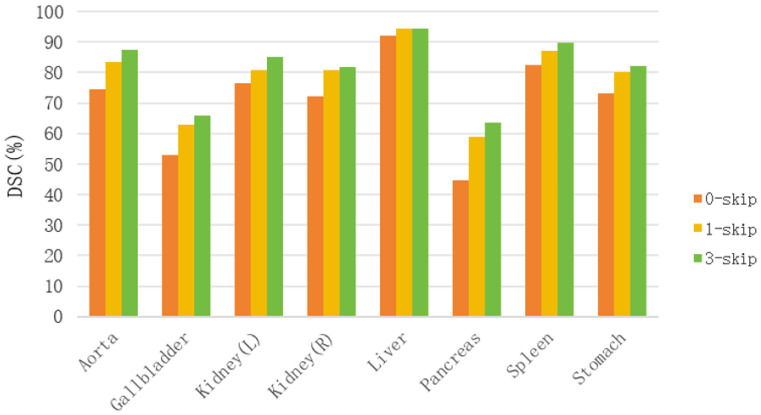
Impact of the number of skip connections on segmentation performance.

Skip connections are critical in medical image segmentation for fusing shallow spatial details with deep semantic features. To validate their efficacy in our architecture, we trained variants with 0, 1, and 3 skip connections. As illustrated in Figure 6, increasing the number of skip connections leads to consistent and significant improvements in DSC across all organs. This confirms that propagating high-resolution features from the encoder to the decoder is essential for alleviating the vanishing gradient problem and recovering fine-grained boundary details.

#### 4.2.2. Impact of Input Resolution

We evaluated the trade-off between performance and computational cost by increasing the input resolution from 224×224 to 512×512. As shown in Table 3 (Top), higher-resolution inputs generally yield better segmentation results. For instance, DSC for the Aorta improves from 88.13% to 91.14%, and the Liver from 93.62% to 95.00%. The average DSC increases to 85.00%, with HD95 improving to 9.92 mm.

However, owing to the quadratic complexity of the self-attention mechanism, the 512×512 resolution significantly increases GPU memory consumption and training time. While it offers superior accuracy, the 224×224 configuration achieves a competitive DSC of 82.79% with much lower resource requirements. Therefore, we adopt 224×224 as the default resolution for a balanced trade-off between accuracy and practical feasibility in clinical settings.

#### 4.2.3. Impact of Patch Size

We analyzed the effect of patch size (P=32,16,8) on model performance (see Table 3 (Middle)). Reducing the patch size from 32 to 8 increases the sequence length from 49 to 784, allowing the model to capture finer spatial details. Consequently, the average DSC improves from 81.78% to 83.45%.

However, this gain comes at a high computational cost, with FLOPs rising from 28.47 G to 41.53 G. Notably, P=8 requires 48% more computation than P=16 but yields only a marginal DSC gain (+0.66%). Conversely, P=32 is efficient but sacrifices accuracy on small organs like the pancreas (DSC drops by 3.33%). We selected 16×16 as the optimal patch size, achieving robust performance (82.79% DSC) with moderate computational overhead (32.08 G FLOPs).

#### 4.2.4. Impact of Model Scale

We compared a “Base” configuration (Hidden Dimension D=768, Layers = 12) against a “Large” configuration (D=1024, Layers = 24). As shown in Table 3 (Bottom), the Large model provides a slight improvement in DSC (83.33% vs. 82.79%). While it benefits fine structures like the pancreas (+3.30%), performance degrades slightly for organs like the left kidney and spleen, potentially due to overfitting or optimization difficulties in deeper networks. Given the significantly higher training cost of the Large model, we utilize the Base configuration for all primary experiments.

#### 4.2.5. Component-Wise Ablation Analysis

To quantify the specific contribution of each proposed module and identify the dominant factors driving performance, we performed a step-by-step ablation study (see Table 4). We started with the original TransUNet as the raw baseline (77.48% DSC, 31.69 mm HD95).

- Foundational Impact of Medical-Specific Augmentation: First, we introduced a data augmentation strategy tailored for medical imaging, specifically incorporating elastic deformations to simulate non-rigid soft tissue distortions. This step proved critical for boundary stability, resulting in a substantial reduction in HD95 from 31.69 mm to 19.33 mm. This establishes a robust baseline, ensuring that subsequent gains are attributed to architectural improvements rather than data scarcity.
- Dominant Contributor to Accuracy (Hybrid Encoder): On top of this augmented baseline, the integration of the enhanced encoder (MSGA + ME-MSA) emerged as the primary driver for segmentation accuracy. It delivered the largest single-module DSC gain (+2.58%, reaching 81.03%), confirming that capturing multi-scale context and spatial priors is fundamental for semantic consistency.
- Critical Contributor to Boundary Precision (MKGAG): The MKGAG module served as the most critical component for further boundary refinement. Even after the strong improvements from augmentation, adding MKGAG reduced the HD95 by an additional 6.58 mm (dropping to 12.75 mm). This confirms that the gating mechanism effectively filters residual background noise that augmentation alone cannot resolve.
- Synergistic Integration: While the Enhanced Decoder (MSSA) refined local details (+2.14% DSC), the peak performance (82.79% DSC, 10.21 mm HD95) was achieved only when all components were integrated, validating the synergistic design of M^3^-TransUNet.

### 4.3. Generalization Experiments

To validate the robustness of M^3^-TransUNet across different modalities, we extended our evaluation to three additional datasets: CVC-ClinicDB (endoscopy), Chest X-ray Masks and Labels (radiography), and ISIC 2016 (dermoscopy).

Experimental Protocol and Robustness: To rigorously assess the model’s inherent generalization capability, we maintained a unified hyperparameter configuration across all datasets without dataset-specific tuning. Specifically, critical parameters including the optimizer (SGD with momentum), initial learning rate, and weight decay were kept consistent with the primary experiments. The only adjustments were restricted to the training epochs and batch sizes to accommodate differences in dataset scale. This protocol ensures that the observed performance gains are derived from the architectural efficacy of M^3^-TransUNet rather than overfitting to specific training configurations.

In addition to DSC and HD95, we employed Intersection over Union (IOU) to provide a more comprehensive assessment of regional overlap:

(18) $$\mathrm{IOU}=\frac{\left|A\cap B\right|}{\left|A\cup B\right|}$$

where *A* and *B* denote the predicted and ground truth regions, respectively.

As summarized in Table 5, M^3^-TransUNet consistently outperforms established baselines (TransUNet, Swin-UNet, DA-TransUNet) across all datasets.

- On CVC-ClinicDB, it achieves the highest DSC (92.26%) and IOU (84.52%), with the lowest HD95 (12.37), indicating accurate segmentation of small polyps despite lighting variations.
- On Chest X-ray, it attains the best DSC (96.36%) and HD95 (11.57%), demonstrating strong capability in delineating lung fields amidst noise.
- On ISIC 2016, it leads in both DSC (91.77%) and IOU (85.42%), confirming its effectiveness in handling irregular lesion boundaries.

Performance Justification on Limited Data: It is worth noting that the high metric values observed (e.g., DSC >96% on Chest X-ray) are consistent with the inherent characteristics of these tasks. For instance, the high contrast between lung fields and surrounding tissues typically yields high baseline scores, as evidenced by TransUNet already achieving 96.10% DSC. Our results represent a competitive incremental improvement rather than an anomaly. The notable gains on the more challenging CVC-ClinicDB dataset are attributed to the hybrid architecture’s ability to model global context while preserving local boundary details, reinforced by the on-the-fly data augmentation strategy (discussed in Section 3.1.2) which prevents overfitting to the limited training samples. These results suggest that M^3^-TransUNet possesses adaptable generalization capabilities for diverse clinical tasks.

### 4.4. Qualitative Visualization and Analysis

**Figure 7:**
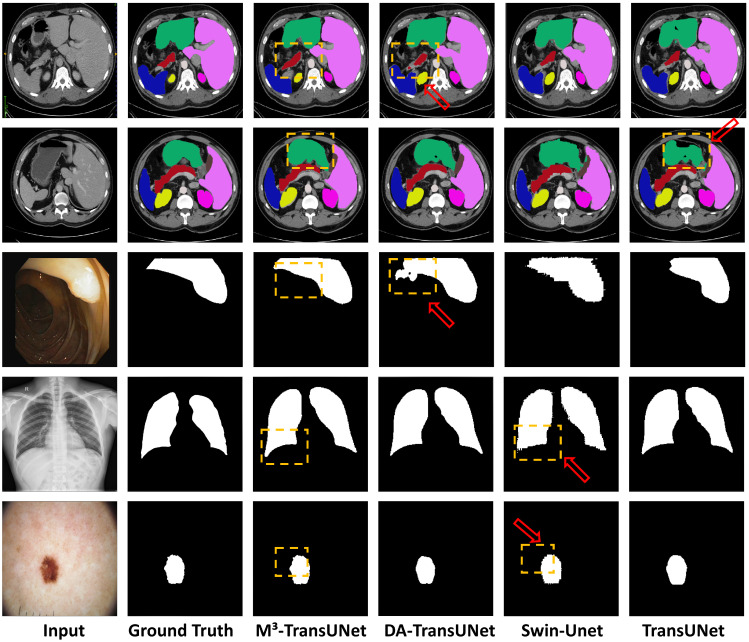
Qualitative comparison of segmentation results across four datasets. The rows correspond to different datasets: (**Rows 1–2:**) Synapse multi-organ segmentation; (**Row 3:**) CVC-ClinicDB polyp segmentation; (**Row 4:**) Chest X-ray lung segmentation; (**Row 5:**) ISIC 2016 skin lesion segmentation. The columns display (left to right): (1) Input Image, (2) Ground Truth, (3) **M^3^-TransUNet (Ours)**, (4) DA-TransUNet, (5) Swin-Unet, and (6) TransUNet. Our method (3rd column) consistently produces sharper boundaries and fewer artifacts compared to the baseline Transformers shown in the rightmost columns.

To provide a direct assessment of the segmentation quality, we present a visual comparison against current state-of-the-art methods. The visualization results across four distinct datasets—Synapse, CVC-ClinicDB, Chest X-ray, and ISIC 2016—are illustrated in Figure 7. To facilitate detailed comparison, specific regions of interest are explicitly highlighted with arrows and yellow dashed boxes.

Impact on Small Structures: As visualized in the first two rows of Figure 7, abdominal organ segmentation presents challenges due to low inter-organ contrast. It can be observed that competing methods in the last three columns (DA-TransUNet, Swin-Unet, and TransUNet) exhibit boundary leakage in soft tissues. Specifically, as indicated by the red arrows, baseline models tend to have difficulty distinguishing the boundaries between the pancreas, stomach, and surrounding organs, leading to potential semantic confusion or under-segmentation. In contrast, our M^3^-TransUNet (3rd column) generates accurate segmentation masks that align well with the Ground Truth. This capability to preserve the topological integrity of small or complex organs is primarily driven by the MSGA module in the encoder, which captures features at multiple receptive fields, ensuring that small targets are not lost during downsampling.

Impact on Low-Contrast Boundaries: For targets with ambiguous boundaries, such as the polyps in the 3rd row and skin lesions in the bottom row, the task is complicated by specular reflections, hair artifacts, and varying lighting conditions. While baseline models may produce fragmented masks or under-segment margins, the regions marked by red arrows indicate that M^3^-TransUNet delineates the complete structure with continuous edges. This benefit stems from the MSSA module in the decoder, which selectively aggregates multi-scale context to refine edge details and suppress background artifacts in low-contrast regions.

Information Preservation and Noise Suppression: Moving to the 4th row for lung segmentation, models must contend with low contrast at the lung bases while preserving fine structural details. M^3^-TransUNet produces cohesive masks with clear delineations at the costophrenic angles. This result improves upon the irregular edges sometimes produced by traditional Transformer-based methods. Crucially, this balance is facilitated by the MKGAG module, which filters noise from the skip connections without over-suppressing useful information. By selectively gating low-level features, the model preserves the smooth anatomical curves of the lungs, as highlighted in the visual comparison, demonstrating that essential spatial details are retained even when background noise is suppressed.

## 5. Discussion

The M^3^-TransUNet framework proposed in this study demonstrates consistent competitive performance across a diverse array of medical image segmentation tasks, ranging from abdominal multi-organ segmentation on Synapse to polyp detection on CVC-ClinicDB, lung segmentation on Chest X-ray, and skin lesion delineation on ISIC 2016. This robustness is primarily attributed to the synergistic design of its core components. Specifically, the MSGA and MSSA modules effectively enhance multi-scale feature representation, enabling the model to adapt to objects of varying sizes. Meanwhile, the ME-MSA incorporates Manhattan distance as a spatial prior, which aligns better with the grid-like structure of medical images compared to Euclidean distance, thereby improving local structural modeling. Furthermore, the MKGAG-based skip connections serve as adaptive gates that suppress irrelevant background noise while preserving fine anatomical details.

Regarding the performance on limited data, it is worth noting that M^3^-TransUNet achieves high segmentation metrics (e.g., 82.79% DSC on Synapse) despite the relatively small scale of training datasets. We attribute this to three synergistic factors. First, the results are consistent with the trajectory of current state-of-the-art benchmarks (e.g., J-CaPA). Second, the hybrid architecture inherently improves sample efficiency; the CNN branch introduces strong inductive biases (locality and translation invariance) which facilitate rapid convergence, while the MKGAG module acts as a regularization mechanism. Third, the extensive on-the-fly data augmentation strategy employed during training prevents the model from overfitting to the limited samples, ensuring that the reported metrics reflect genuine generalization capability.

Despite these achievements, we acknowledge several limitations inherent to the proposed hybrid paradigm. First, regarding data requirements, while the CNN branch introduces helpful inductive biases, the Transformer component remains fundamentally data-hungry. Our high performance relies significantly on the aforementioned online data augmentation; without such regularization, the risk of overfitting due to the high parameter count (116.95 M) remains a concern. Second, regarding training stability, the joint optimization of heterogeneous architectures is non-trivial. Although our analysis confirms stable convergence, hybrid models generally exhibit higher sensitivity to hyperparameter settings compared to standard CNN baselines. Finally, as observed in qualitative visualizations, pixel-level refinements remain challenging under extreme lighting conditions (e.g., polyp margins) or within low-contrast soft tissues, indicating room for further improvement in boundary robustness.

## 6. Conclusions

In this paper, we introduced M^3^-TransUNet, a novel hybrid segmentation architecture designed to address critical bottlenecks in medical image analysis. By integrating multi-scale gated attention, spatial-aware self-attention with Manhattan priors, and noise-filtering skip connections, our approach effectively balances global semantic understanding with local detail preservation. Extensive experiments on four benchmark datasets validate its competitiveness against existing CNN and Transformer-based methods in terms of segmentation accuracy and generalization capability.

Future work will focus on two key directions to address the identified limitations. First, we aim to develop a streamlined variant of M^3^-TransUNet through techniques such as knowledge distillation or structural pruning, facilitating real-time deployment in clinical settings. Second, we plan to extend the framework to 3D segmentation by incorporating inter-slice attention mechanisms, allowing the model to leverage full volumetric spatial context for improved consistency in tomographic imaging.

## Figures and Tables

**Table 1:** Model Performance Comparison on the Synapse Dataset. The symbol ‘*’ indicates statistical significance (p<0.05) compared to the second-best method using the Wilcoxon signed-rank test.

Model	Aor.	Gal.	LKid.	RKid.	Liv.	Pan.	Spl.	Sto.	DSC	HD95
U-Net	89.07	69.72	77.77	68.60	93.43	53.98	86.67	75.58	76.85%	39.70
U-Net++	88.19	68.89	81.43	75.27	93.01	58.20	83.44	70.52	76.91%	36.93
Residual U-Net	87.06	66.05	83.43	76.83	93.99	51.86	85.27	70.13	76.95%	38.06
Att-UNet	89.55	68.88	77.98	71.11	93.57	58.04	87.30	75.75	77.77%	36.02
MultiResUNet	87.73	65.67	82.08	70.43	93.49	60.09	85.23	75.66	77.42%	36.84
TransUNet	87.23	63.13	81.87	77.02	94.08	55.86	85.08	75.62	77.48%	31.69
UCTransUNet	84.25	64.65	82.35	77.65	94.36	58.18	84.74	79.66	78.23%	26.75
TransNorm	86.23	65.10	82.18	78.63	94.22	55.34	89.50	76.01	78.40%	30.25
Swin-Unet	85.47	66.53	83.28	79.61	94.29	56.58	90.66	76.60	79.13%	21.55
DA-TransUNet	86.54	65.27	81.70	80.45	94.57	61.62	88.53	79.73	79.80%	23.48
HiFormer-B	86.21	65.23	85.23	79.77	94.61	59.52	90.99	81.08	80.39%	14.70
PVT-VASVADE	83.01	70.59	82.23	80.37	94.08	64.43	90.10	83.69	81.06%	20.23
J-CaPA	88.28	63.10	86.00	83.10	94.60	69.30	90.70	83.30	82.29%	19.74
**Ours**	88.13	70.24	86.37	83.08	93.62	68.45	90.88	82.21	82.79% *	10.21 *

**Table 2:** Comparison of model complexity and efficiency on the Synapse dataset. The best performance is bolded.

Method	Params (M)	FLOPs (G)	DSC (%)
U-Net	34.52	65.52	76.85
Att-UNet	34.88	66.60	77.77
TransUNet	105.32	38.52	77.48
Swin-UNet	27.14	5.91	79.13
HiFormer-B	25.51	8.05	80.39
PVT-CASCADE	35.28	6.40	81.06
MISSFormer	35.45	7.25	81.96
J-CaPA	-	-	82.29
**M^3^-TransUNet (Ours)**	116.95	32.08	82.79

**Table 3:** Ablation studies on Input Resolution, Patch Size, and Model Scale.

Setting	FLOPs	Aor.	Gal.	LKid.	RKid.	Liv.	Pan.	Spl.	Sto.	DSC	HD95
(a) Impact of Input Resolution											
224×224	-	88.13	70.24	86.37	83.08	93.62	68.45	90.88	82.21	82.79	10.21
512×512	-	91.14	72.12	86.58	84.59	95.00	73.41	90.93	86.18	85.00	9.92
(b) Impact of Patch Size (Resolution fixed at 224)											
Patch = 32	28.47 G	87.12	69.23	85.36	82.07	92.61	67.44	89.87	81.20	81.78	11.01
Patch = 16	32.08 G	88.13	70.24	86.37	83.08	93.62	68.45	90.88	82.21	82.79	10.21
Patch = 8	41.53 G	89.59	70.57	85.03	83.05	93.46	71.87	89.39	84.64	83.45	10.01
(c) Impact of Model Scale (Patch = 16, Resolution = 224)											
Base	-	88.13	70.24	86.37	83.08	93.62	68.45	90.88	82.21	82.79	10.21
Large	-	89.48	70.46	84.92	82.93	93.34	71.75	89.27	84.52	83.33	10.04

**Table 4:** Step-by-step ablation study of enhancement modules.

Model Variant	MKGAG	MSSA	Encoder+	DSC (%)	HD95 (mm)
Baseline (TransUNet)	-	-	-	77.48	31.69
Baseline + Aug	-	-	-	78.45	19.33
+ Enhanced Skip	✔	-	-	79.53	12.75
+ Enhanced Decoder	-	✔	-	80.59	11.84
+ Enhanced Encoder	-	-	✔	81.03	11.48
**M^3^-TransUNet (Ours)**	✔	✔	✔	82.79	10.21

**Table 5:** Generalization performance on CVC-ClinicDB, Chest X-ray, and ISIC 2016 datasets.

Model	CVC-ClinicDB			Chest X-ray			ISIC 2016		
	**DSC**	**HD95**	**IOU**	**DSC**	**HD95**	**IOU**	**DSC**	**HD95**	**IOU**
TransUNet	84.91	26.14	77.45	96.10	11.81	92.58	91.03	73.31	84.32
Swin-UNet	86.39	23.33	78.27	95.10	15.20	90.73	91.37	58.44	84.62
DA-TransUNet	88.62	23.68	82.20	96.20	12.54	92.75	91.65	60.44	85.32
**M^3^-TransUNet**	92.26	12.37	84.52	96.36	11.57	92.38	91.77	59.51	85.42

## Data Availability

The original contributions presented in this study are included in the article. Further inquiries can be directed to the corresponding author.
